# Integrated Process for the Enzymatic Production of Fatty Acid Sugar Esters Completely Based on Lignocellulosic Substrates

**DOI:** 10.3389/fchem.2018.00421

**Published:** 2018-09-13

**Authors:** Sascha Siebenhaller, Jennifer Kirchhoff, Frank Kirschhöfer, Gerald Brenner-Weiß, Claudia Muhle-Goll, Burkhard Luy, Fabian Haitz, Thomas Hahn, Susanne Zibek, Christoph Syldatk, Katrin Ochsenreither

**Affiliations:** ^1^Institute of Process Engineering in Life Sciences, Section II: Technical Biology, Karlsruhe Institute of Technology, Karlsruhe, Germany; ^2^Institute of Functional Interfaces, Karlsruhe Institute of Technology, Karlsruhe, Germany; ^3^Institute of Organic Chemistry and Institute for Biological Interfaces 4, Karlsruhe Institute of Technology, Karlsruhe, Germany; ^4^Fraunhofer Institute for Interfacial Engineering and Biotechnology, Stuttgart, Germany

**Keywords:** *Cryptococcus curvatus*, single cell oil, lignocellulose, sugar esters, synthesis, deep eutectic solvents, biorefinery

## Abstract

Lignocellulose can be converted sustainably to fuels, power and value-added chemicals like fatty acid esters. This study presents a concept for the first eco-friendly enzymatic synthesis of economically important fatty acid sugar esters based on lignocellulosic biomass. To achieve this, beech wood cellulose fiber hydrolysate was applied in three manners: as sugar component, as part of the deep eutectic solvent (DES) reaction system and as carbon source for the microbial production of the fatty acid component. These fatty acids were gained from single cell oil produced by the oleaginous yeast *Cryptococcus curvatus* cultivated with cellulose fiber hydrolysate as carbon source. Afterwards, an immobilized *Candida antarctica* lipase B was used as the biocatalyst in DES to esterify sugars with fatty acids. Properties of the DES were determined and synthesized sugar mono- and di-esters were identified and characterized using TLC, MS, and NMR. Using this approach, sugar esters were successfully synthesized which are 100% based on lignocellulosic biomass.

## Introduction

With an estimated production of 120–140 billion tons per year, lignocellulose constitutes the most abundant organic material in the world. Lignocellulose is the main component of woody and grass-like biomass and consists of cellulose, hemicellulose and lignin, containing up to 75% of carbohydrates and therefore, it is excellently suited as a substrate for a biorefinery (Herrera, 2006; Pauly and Keegstra, 2008; Zhao et al., 2009; Cherubini, 2010; FitzPatrick et al., 2010). In contrast to renewable resources of the first generation, these carbohydrates are not competing with food production. They are a valuable substrate for both biotechnological and (bio-)chemical processes but require previous fractionation. By applying the physico-chemical organosolv process the three main components from lignocellulose can be separated using an aqueous-organic solvent system under high pressure and temperature. The cellulose fiber fraction can be subsequently saccharified enzymatically yielding a glucose-rich hydrolysate, which is highly suitable as a microbial carbon source, as a building block or as a pre-cursor for other (fine-)chemicals (Nigam, 2001; Laure et al., 2014; Dörsam et al., 2017).

Surfactants are amphiphilic molecules, which occur in numerous products of daily life in food, cosmetics and pharmaceuticals (Šabeder et al., 2006; Marchant and Banat, 2012). Fatty acid sugar esters (a.k.a. glycolipids) are a class of surfactants, based on a sugar moiety and a fatty acid tail. Compared to other surfactant classes, they are commonly biodegradable, non-toxic and environmentally friendly being very mild and skin-friendly at the same time (Chang and Shaw, 2009). Synthesis of sugar esters can be performed by chemical processes as well as by microbial or enzymatic conversions (Ducret et al., 1996). However, only enzymatic synthesis enables sustainable production of tailor-made sugar esters. Enzymatic synthesis of fatty acid sugar esters based on sugar and fatty acids or their respective esters is often catalyzed by esterases or lipases in reaction media of low water activity (Pöhnlein et al., 2015). Under these conditions, the hydrolytic activity of both enzyme classes is reversed and ester bonds are formed between the hydroxyl group of a sugar molecule and the carboxyl group of the fatty acid (Zaks and Klibanov, 1985; Klibanov, 1989). Esterification of sugar and fatty acid can take place in different reaction media, e.g., certain organic solvents, ionic liquids or deep eutectic solvents (DES). DES offer several advantages, as non-flammable, cheap, biodegradable and easy to produce. In general, DES consists of a mixture of a quaternary ammonium or phosphonium salt and a hydrogen bond donor. By interaction of both components the melting point of the mixture is strongly decreased resulting in a solution which is often liquid at room temperature (Abbott et al., 2002; Durand et al., 2012; Tang and Row, 2013). Since it was shown by Gorke et al. (2008), that hydrolases like esterases and lipases show high activity in deep eutectic solvents consisting of the ammonium salts choline chloride (CC) or ethylammonium chloride with different hydrogen bond donors, many other successful enzymatic reactions in DES were reported (Xu et al., 2017).

In a previous study it was shown, that various sugars can act simultaneously as hydrogen bond donor for the DES formation, and as substrate for the enzymatic sugar ester production (Siebenhaller et al., 2016). Further, it was shown, that fatty acid vinyl esters with different chain length are suitable acyl chains. Siebenhaller et al. (2017) demonstrated that it is possible to use a cellulose fiber hydrolysate from beech wood as a sugar resource for both, enzymatic reaction and DES component, to synthesize sugar esters with a fossil-oil based fatty acid tail. To increase sustainability of the product, a renewable source for fatty acids or fatty acid esters like plant oil or microbial oil (“single cell oil,” SCO) can be used. In contrast to plant oils, microbial lipids can be produced independent from season, climate and location; they do not require arable land and a wide range of carbon sources, e.g., waste streams from food industry or renewable carbon sources, can be used for their production (Ochsenreither et al., 2016). SCOs are produced by oleaginous microorganisms (bacteria, yeast, microalgae or filamentous fungi) which are able to accumulate 20–80% lipid per dry biomass in the stationary growth phase under nutrient limitations, e.g., nitrogen or phosphate, and coincident excess of carbon source (Ratledge, 2004). The basidiomycetous yeast *Cryptococcus curvatus* belongs to one of the best studied oleaginous microorganisms. Under optimized conditions, *C. curvatus* accumulates up to 60% lipid per dry biomass (Ratledge, 1991). Previous studies showed that *C. curvatus* can utilize waste streams from industry, e.g., distillery or brewery waste, to produce SCO (Gonzales-Garcia et al., 2013; Ryu et al., 2013). Additionally, some hydrolysates, such as dilute sulfuric acid hydrolysates of wheat straw (with emphasis on acetate utilization) (Yu et al., 2014) and alkaline-pretreated corn stover hydrolysate (resulting in ~30% (w/w) lipid content) (Gong et al., 2015) have been used as a substrate.

However, to the best of our knowledge, enzymatically saccharified beech wood cellulose fiber has not been studied so far as a suitable carbon source for lipid production by *C. curvatus*.

The aim of the presented concept was to produce fatty acid sugar esters from completely renewable resources using eco-friendly enzymatic catalysis. The choice of DES as a reaction medium offers a green alternative and therefore characteristics of the solvent were determined. In this system, the synthesis of sugar esters can be carried out using the immobilized *Candida antarctica* lipase B (iCalB). The successful formation was proven by NMR and MALDI-ToF analysis. This set-up, together with sugars and fatty acids based on beech wood cellulose fiber hydrolysate, enables the production of surfactants of 100% renewable origin.

## Materials and methods

### Preparation of beech wood cellulose fiber hydrolysate

Chopped beech wood was incubated at temperatures >140°C in aqueous ethanol solution with a small amount of H_2_SO_4_ as catalyst to obtain different fractions (acid-catalyzed organosolv process). After washing, the fiber fraction, mainly containing the cellulose and a part of hemicellulose, was directly subjected to enzymatic hydrolysis. Enzymatic hydrolysis of the fiber was performed for 24 h at a temperature of 50°C with a 10% (w/v) suspension. For stirrer description see Ludwig et al. (Ludwig et al., 2014). pH of the suspension was adjusted to pH 4.8 during hydrolysis using a concentrated NaOH solution. The hydrolysis reaction started by adding 60 mg Cellic® CTec3 and 2.5 mg Cellic® HTec3 per g cellulose. Solid material was removed afterwards applying an extruder press. The successive concentration of the filtrate resulted in the following mono- and disaccharide concentrations: 30.8 g/L cellobiose, 89.7 g/L xylose and 608.3 g/L glucose quantified via chromatographic analysis (see Sluiter et al., 2008 for further description). Analysis furthermore revealed the absence of toxic or degradation products.

The obtained cellulose hydrolysate was directly used as carbon source for SCO production. For the preparation of DES, however, it had to be further purified and dried: The cellulose hydrolysate was diluted in ddH_2_O (1:2 w/v) and 1 g activated carbon per 5 mL solution was added. After rigorous shaking for 1 min, the activated carbon mixture was incubated for 3 h at room temperature. Succeeding purification, activated carbon was separated by a two-step filtration process (pore size 4–7 and 0.22 μm). Afterwards, the solution was spray dried in a werco® SD-20 spray dryer (input temperature 182°C, output temperature 74–81°C; Hans G. Werner Industrietechnik, Reutlingen, Germany) resulting in a final water content of 8%, determined by Karl Fischer titration (TitroLine® 7500 KF trace, SI Analytics, Mainz, Germany). Mono- and disaccharide concentrations were analyzed by HPLC (see section Analysis of Bioreactor Samples).

### Production of single cell oil in a bioreactor

#### Strain and medium

For the production of single cell oil, *C. curvatus* ATCC 20509 was used. Cultivation was conducted as outlined by Dillschneider et al. (2014). Briefly, the culture medium was based on a phosphate buffer pH 5 (8.99 g/L KH_2_PO_4_ and 0.12 g/L Na_2_HPO_4_ · 2 H_2_O). The medium constituents were 0.1 g/L sodium citrate (C_6_H_5_O_7_Na_3_ · 2 H_2_O), 0.1 g/L yeast extract, 0.2 g/L MgSO_4_ · 7 H_2_O and 4.72 g/L (NH_4_)_2_SO_4_. Medium was supplemented with 2 mL trace elements solution (4 g/L CaCl_2_ · 2 H_2_O, 0.55 g/L FeSO_4_ · 7 H_2_O, 0.475 g/L citric acid, 0.1 g/L ZnSO_4_ · 7 H_2_O, 0.076 g/L MnSO_4_ · H_2_O, 100 μL18 M H_2_SO_4_) and 1 mL salt solution (20 g/L MgSO_4_ · 7 H_2_O, 10 g/L yeast extract) per 100 mL cultivation medium. During cultivation, salts and trace elements were added once every 24 h to the culture broth. Unpurified cellulose fiber hydrolysate was used as carbon source. The hydrolysate was diluted with distilled water to obtain a stock solution with a concentration of 500 g/L carbohydrates. The initial sugar concentration in the culture medium was 50 g/L. Stock solution was fed daily to keep sugar concentration constant between 50 and 90 g/L during the whole cultivation.

#### Cultivation and sampling

The first pre-culture was prepared in 20 mL medium in 100 mL conical shake flasks and was inoculated with 100 μL glycerol stock culture of *C. curvatus* (15% w/w, stored at −80°C). The second pre-culture with 200 mL culture medium in 2 L shake flasks was inoculated with the first pre-culture to obtain an initial OD_600_ of 1. Both pre-cultures were incubated at 28°C with 120 rpm for ~24 h.

Fermentation of the main-culture was performed in duplicate in a 2.5 L bioreactor (Infors HT, Bottmingen, Switzerland; Minifors fermentor) with 1.2 L culture medium at the beginning, initial OD_600_ of 1, at 600 rpm, 28°C and with 1 vvm aeration rate for 96 h. Stirrer speed was increased to 800 rpm when dissolved oxygen concentration decreased below 15%. The pH was adjusted to 5.0 by controlled addition of 4 M H_3_PO_4_ and 4 M NaOH. Contraspum A 4050 HAC (Zschimmer und Schwarz) was applied as antifoam agent.

**Figure 1:**
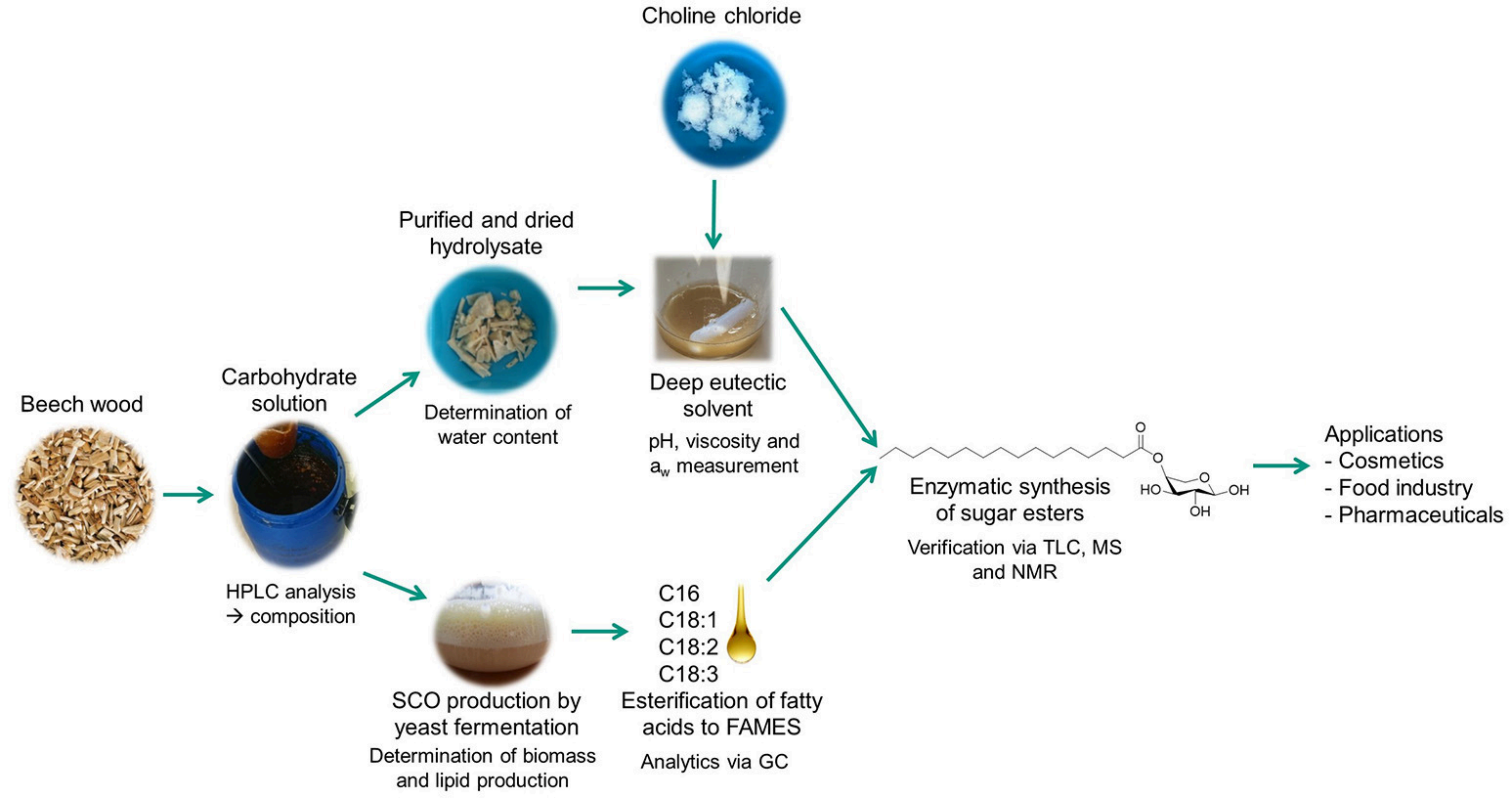
Flowchart of the study. Carbohydrates from beech wood cellulose fiber hydrolysates were used to produce fatty acids in a yeast bioprocess, to form the solvent system and as substrate for the enzymatic reaction to produce fatty acid sugar esters (a.k.a glycolipids). Analytical methods are indicated at each work package.

Each day 4 samples were taken as given in Figure 1. Prior to sampling, 20 mL of culture broth was discarded. Sampling volume was 5 mL (three samples) or 20 mL (one sample). Samples were subjected to OD_600_, dry biomass and sugar concentration measurement. Lipid content and composition were only determined in the 20 mL sample, ammonium content only in the first samples until complete consumption. Feeding of salts, trace elements and carbon source was done 30 min before taking the last sample of the day.

#### Single cell oil extraction and transesterification

The yeast biomass of each 20 mL sample and the complete biomass in the bioreactor at the end of the cultivation was collected by centrifugation (5 min, 4,600 × g), washed once in sterile saline and pelleted again by a second centrifugation. Supernatants were discarded and the resulting cell pellet was immediately frozen in liquid nitrogen. Biomass was freeze-dried for at least 24 h, at −30°C and 0.37 mbar.

For analytical purposes and fatty acid sugar esters synthesis, fatty acid methyl esters were formed by transesterification from single cell oil as described below. For lipid analysis, 20 mg of freeze-dried biomass was incubated for 2 h at 100°C in a thermoshaker at 1,400 rpm in the presence of 1.5 mL n-hexane, 2 mL 15% H_2_SO_4_ in methanol and 0.5 mL of an internal standard (2 mg/mL methyl benzoate). After cooling on ice, 1 mL demineralized water was added. The mixture was centrifuged for 5 min at 2,500 rpm. 1 μL of the upper phase, containing the fatty acid methyl esters extract, was analyzed via gas chromatography.

For the production of sugar esters, 1 g of freeze-dried biomass was transesterified in the presence of 25 mL n-hexane and 25 mL 15% H_2_SO_4_ in methanol for 5 h at 90°C in an oil-bath under constant stirring (350 rpm). After cooling on ice, 5 mL demineralized water was added. The upper organic phase was collected and solvent in this phase was evaporated at 40°C and 2,000 rpm in a speed vac. The produced fatty acid methyl esters were used as substrate for sugar ester synthesis.

#### Analysis of bioreactor samples

Dry biomass was analyzed gravimetrically. 1 mL of the culture broth was transferred into a pre-dried and pre-weighed reaction tube and centrifuged at 13,000 rpm for 5 min. The supernatant was collected and used for the determination of sugar and ammonia content. The cell pellet was washed with saline, dried at 60°C for 24 h and weighed.

Ammonium concentration was determined enzymatically using Spectroquant kit (Merck KGaA, Darmstadt, Germany) following the instructions of the manufacturer.

For the carbohydrate quantification with HPLC, fermentation broth samples were pre-treated and analyzed as described by Buchholz et al. (2013) with slight modifications. A protocol for phosphate precipitation was applied before measurement. 1 mL of culture supernatant was mixed with 45 μL 4 M NH_3_ and 100 μL 1.2 M MgSO_4_, incubated for 5 min at RT and subsequently centrifuged for 5 min at 13,000 rpm. 500 μL of the resulting supernatant was then mixed with 500 μL 0.1 M H_2_SO_4_, incubated for 15 min followed by centrifugation for 15 min at 13,000 rpm. The supernatant was used for HPLC analysis of the carbohydrates as described by Siebenhaller et al. (2018). Calibration with pure glucose and xylose ranged from 10 to 500 mg/L.

Lipid content and composition were determined by analyzing the fatty acid methyl esters, obtained by transesterification, via GC (Agilent Technologies, 6890 N Network GC-System). The instrument was equipped with a DB Wax column (l: 30 m; d: 0.25 mm, Agilent Technologies Deutschland GmbH, Böblingen, Germany), and a flame ionization detector. The working pressure was 1.083 bar and initial temperature 40°C. The column temperature was increased from 40° to 250°C with a rate of 8°C/min. The temperature was maintained at 250°C for 20 min before cooling down to 40°C. Fatty acids were identified and the total fatty acid content was determined with the standard FAME Mix RM-3 (Supelco).

### Enzymatic synthesis of fatty acid sugar esters

#### Preparation and evaluation of the deep eutectic solvent

The purified and dried cellulose hydrolysate fraction consists of 71.6% glucose as well as 16.6% xylose and was mixed with choline chloride in a ratio of 1:1 (w/w). Afterwards, the mixture was stirred and heated up to 100°C until the liquid DES was formed. The DES was directly used as solvent and substrate for the enzymatic sugar ester synthesis.

DES viscosity was determined at 25° and 50°C with a rheometer (5 cm plate diameter, 1 mm gap, shear rate 0–222 [1/s]; Physica MCR 301, Anton Paar GmbH, Austria). The water activity was measured of a fresh DES with a LabMaster-aw neo (Novasina AG, Switzerland).

#### Synthesis reaction and extraction of fatty acid sugar esters

Sugar esters were synthesized in 5 mL Eppendorf cups by adding 100 mg of iCalB (Lipase acrylic resin from *C. antarctica*, Sigma-Aldrich, Taufkirchen, Germany) to 2.5 mL of DES. To start the reaction, 175 μL of the prepared fatty acid methyl esters were added and the reaction was performed at 50°C for 70 h in a rotator with vortex mixer (neoLab, Heidelberg, Germany) in program U2 at 50 rpm. As controls, reactions without enzyme or without fatty acids were performed.

To stop the reaction, 2 mL of warm water was added and the mixture was shaken until full dissolution of the DES. Organic phase extraction with the same volume of ethyl acetate resulted in the accumulation of sugar esters after 45 s of mixing. The sugar ester containing organic phase was removed for further experiments. To enhance extraction efficiency, the addition of ethyl acetate with subsequent mixing can be repeated up to five times.

### Purification of fatty acid sugar esters by flash chromatography

Before further analysis via mass spectrometry and NMR, eight extracts from identical synthesis reactions were unified and purified via flash chromatography (Reveleris Prep, Büchi Labortechnik GmbH, Germany). The extract was mixed with 2 g of silica (40 μm pore size) and liquid was subsequently evaporated in a rotary evaporator. The silica including the bound components (products and excess of sugar and FAMEs) were packed in an empty column. For separation, a Reveleris HP Silica 4 g column and a flow rate of 15 mL/min was used. As method, the dry sample function was selected and a gradient of chloroform and methanol was used as follows: 0–7% methanol in 1 min, holding the gradient for 7 min, followed by an increase to 15% methanol in 2 min and holding it for 1 min with subsequent increase to 20% in 1 min. To eluate sugars and other polar components, the gradient was set to 100% methanol in 1 min holding it for 4 min. Peaks were observed by an evaporative light scattering detector (Threshold: 30 mV, Sensitivity: low) and fractions collected. Unprocessed fractions were analyzed via TLC and divided into three samples (1 = fraction 6–9, 2 = fraction 14+15+17, 3 = fraction 16). For ESI-Q-ToF MS, MALDI-ToF MS and NMR analysis the samples were evaporated.

### Analysis of fatty acid sugar esters

#### Qualitative detection of fatty acid sugar esters via thin-layer chromatography

As a fast qualitative detection method of the formed sugar esters, 10 μL extracts were spotted onto a 60 Å silica gel TLC plate (Alugram Xtra SIL G, Macherey-Nagel GmbH & Co.KG, Düren, Germany). Compounds were separated using a mobile phase consisting of chloroform: methanol (70: 5, by vol.). Visualization was accomplished by dipping the TLC plate into an anise aldehyde solution (anise aldehyde: sulfuric acid: acetic acid 0.5: 1: 100, by vol.) with subsequent heating under a 200°C air flow for 5 min.

#### Determination of masses via thin-layer chromatography coupled matrix-assisted laser desorption/ionization time of flight

With the TLC-MALDI-ToF technique it is possible to determine the mass of the product spots on a TLC plate. Two identical replicates of a HPTLC plate (5 × 7.5 cm HPTLC plate, silica gel 60, Merk, Darmstadt, Germany) were thus prepared. Ten to fifteen microliter of the purified and concentrated sample 1 and sample 2 from flash chromatography were sprayed as 6 mm horizontal bands on the TLC plate using an automatic sample application device (ATS4, CAMAG, Switzerland) and were separated with the solvent system chloroform: methanol (70: 10, by vol.). One plate was dyed with an anise aldehyde solution. On the second plate, a drop of an external polymer standard (Polypropylenglycol M_N_ 725 (Sigma-Aldrich); 1:100 with methanol) was placed in a corner, afterwards the plate was dipped for 2 s in a matrix solution (200 g/L dihydroxybenzoic acid in 90% acetonitrile, 1.15 g/L diammonium phosphate, 0.2 g/L octyl β-D-glucopyranoside, 0.1% trifluoroacetic acid). Subsequently, the plate was fixed onto the MTP TLC Adapter for a Bruker Ultraflex II ToF/ToF (Bruker AG, Rheinstetten, Germany). The dyed plate served as a template providing a coordinate system for the laser pulses (25 kV ion source, offset 63%, range 15%, positive mode). Every 0.6 mm of the vertical coordinate system, measurements were made at seven juxtaposed positions. The measured dataset was analyzed by the instrument software flexControl 3.0.

#### Sample preparation and MALDI-ToF mass spectrometry

Sample 1 from flash purification were mixed with MALDI matrix solution (ratio 50: 50). The matrix solution consisted of 10 mg/mL α-cyano-4-hydroxycinnamic acid (Sigma–Aldrich, Taufkirchen, Germany) in water/acetonitrile (50: 50, by volume) with 0.2% (volume ratio) trifluoroacetic acid. 1 μL of the mixture was then spotted on the MALDI target and air-dried.

MALDI-ToF MS experiments were carried out on a 4800 MALDI-ToF mass spectrometer (Applied Biosystems/MDS SCIEX, Foster City, CA) in reflector positive ion mode (focus mass 500 Da). Each mass spectrum was an average of 1.000 laser shots over the entire spot. Mass calibration was performed using a calibration solution (Sigma-Aldrich) spotted nearby the samples.

#### Verification of the accurate masses via electrospray ionization quadrupole time of flight mass spectrometry (ESI-Q-ToF MS)

Accurate masses of synthesized and purified fatty acid sugar esters from sample 2 were determined with an ESI-Q-ToF MS system (Q-Star Pulsar i, AB SCIEX, Darmstadt, Germany) equipped with an electrospray ionization (ESI) source. Measurements were accomplished as described in Siebenhaller et al. (2017). Data acquisition and processing were performed using the Analyst QS 1.1 software (AB SCIEX, Darmstadt, Germany).

#### Structural elucidation via nuclear magnetic resonance spectroscopy

For NMR spectroscopy of formed sugar esters, 30 mg of purified sample 3 from the flash chromatography was dissolved in 0.6 mL CDCl_3_/d6-acetone (4: 1, by vol.). 1D ^1^H NMR spectroscopy and 2D ^1^H-^1^H correlation spectroscopy (COZY), ^1^H-^13^C heteronuclear single quantum coherence spectroscopy (clip-HSQC Enthart et al., 2008), and heteronuclear multiple-bond correlation spectroscopy (HMBC) were performed as described in Siebenhaller et al. (2016).

## Results and discussion

For the sustainable production of fatty acid sugar ester biosurfactants monosaccharides originated from enzymatically hydrolyzed cellulose fiber fraction and fatty acid methyl esters derived from microbially produced triacylglycerols (single cell oil) are esterified. In a first step, the production of single cell oil based on cellulose fiber fraction and their transesterification to fatty acid methyl ester (FAME) is reported (3.1). For the enzymatic esterification the DES reaction system consisting of cellulose fiber monosaccharides and choline chloride was prepared and characterized (3.2). Besides as part of the solvent, these monosaccharides act also as substrate for the enzymatic biosurfactant production and are linked to the added FAMEs by iCalB lipase. The successful formation of sugar esters were qualitatively proven by TLC (3.3), mass analysis (3.4) and NMR (3.5). The flowchart of this study is given in Figure 1.

### Production of single cell oil using cellulose fiber hydrolysate

**Figure 2:**
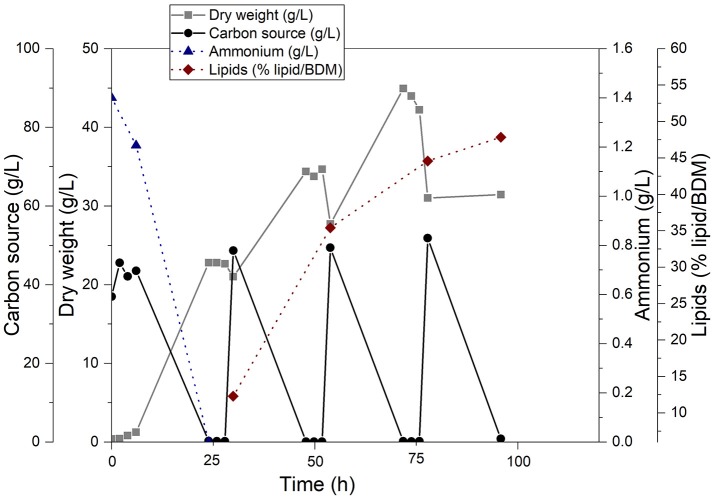
Production of single cell oil with the oleaginous yeast *Cryptococcus curvatus* ATCC 20509 in a 2.5 L bioreactor using cellulose fiber hydrolysate from beech wood. All concentrations are given as averages of two independent bioreactor cultivations. The individual values can be found in Supplement 1.

The fatty acid component for the synthesis of sugar esters was gained from single cell oil produced by *C. curvatus* with cellulose fiber hydrolysate as substrate. Bioreactor cultivation was conducted in duplicate and the time course is shown in Figure 2 as mean of both cultivations.

Cellulose fiber hydrolysate proved to be a very suitable carbon source for both biomass and lipid production. Biomass content increased from 0.4 g/L dry biomass to ~45 g/L dry biomass after 72 h of cultivation. After each addition of carbohydrates, a steep increase of biomass was observed. During carbon source consumption, dissolved oxygen concentration dropped quickly (data not shown) until the carbon source was depleted. Since carbon source was limited between feeding cycles, biomass concentration stagnated or was slightly reduced due to sampling and the dilution of culture broth because of feeding. After feeding at 79 h, biomass dropped considerably and did not increase again. Probably due to the high lipid content of the cells at that time, the cell pellet was very soft and cells started to float. Therefore, solid-liquid separation by centrifugation was not efficient and dry biomass concentration might be higher than given in Figure 2 at the end of cultivation.

Accumulation of single cell oil started after nitrogen depletion at ~24 h of cultivation time. Lipid content increased from ~12% (w/w) to ~48% (w/w) of dry biomass after 96 h showing the steepest increase between 30 and 60 h of cultivation time. The final fatty acid profile analyzed via GC revealed a composition of 44.6% oleic acid (C18:1), 35.1% palmitic acid (C16:0), 12.6% stearic acid (C18:0) and 6.2% linoleic acid (C18:2). Myristic acid (C14:0) has been detected only in trace amounts (0.77%); all other fatty acids add up to 0.74%. In total, 256.5 g of carbohydrates have been consumed during the cultivation.

The successful production of single cell oil with *C. curvatus* based on beech wood cellulose fiber hydrolysate is one of the major outcomes of this study and was shown for the first time. The used fiber hydrolysate consists of a carbohydrate mixture with glucose (608.3 g/L), xylose (89.7 g/L) and cellobiose (30.8 g/L) as the major components. Glucose is likely the most preferred carbon source of *C. curvatus*, however, xylose and cellobiose did not accumulate in culture medium and were completely consumed before the next feeding. The ability of *C. curvatus* to consume xylose and cellobiose as well as a mixture of these two carbon sources with glucose has already been demonstrated by Yu et al. (2014). They demonstrated that cellobiose and xylose can be consumed simultaneously with similar rates, but their consumption is inhibited in the presence of glucose. In a mixture of all three sugars (in pure form) the composition of the mixture had only little impact on the total sugar consumption rate. However, the consumption rate of glucose was considerably higher than the consumption rates of both other sugars. In the presence of a low glucose concentration, i.e., 10 g/L and lower, consumption of xylose and cellobiose seemed to be not affected (Yu et al., 2014). Due to the sampling interval in our study, the exact threshold concentration of glucose cannot be confirmed. However, in preliminary studies in shake flasks it was observed, that xylose accumulated in the medium because glucose was not exhausted before feeding (results not shown). In the study of Yu et al. (2014) the composition of the carbon source did not significantly affect lipid content and composition. Lipid content ranged in all approaches from 38.7 to 40.3% lipid per dry biomass with oleic acid being the main fatty acid (41.3–44.8%) which resembles our results very closely.

Although production of single cell oil is generally possible with all kinds of sugars and various other carbon sources, yields and productivity are usually best when using pure glucose. Therefore, the cultivation with glucose is also used as benchmark for this study. Compared to cultivations with pure glucose as described by Dillschneider et al. (2014), the results of the present study concerning lipid content (~48% in this study vs. 45.3% (w/w)) is very similar. However, concerning the lipid composition (oleic acid: 44.6% in this study vs. 48.8%; linoleic acid: 6.2 in this study vs. 8.6%; palmitic acid: 35.1% in this study vs. 18.5% and stearic acid 12.6% in this study vs. 17.7%), a higher content of palmitic acid was produced at the expense of oleic acid, linoleic acid, stearic acid and lignoceric acid, which was not detected in the presented study. The prominent shift in the fatty acid profile toward C16:0 compounds might be explained due to the use of carbohydrate mixtures in our study compared to pure glucose in the study of Dillschneider et al. (2014). However, this is not supported by the study of Yu et al. (2014) as they also used different carbohydrate mixtures and did only observe a very slight shift in fatty acid profile. Another possible reason might be the presence of by-products in the cellulose fiber fraction due to the organosolv pretreatment, e.g., furfural or HMF. These compounds can be detoxified by the cells, but the mechanisms require the coenzymes NADH or NADPH (Jönsson et al., 2013). For SCO production, high amounts of NADPH are needed, for each addition of a C2 unit 2 NADPH molecules have to be spent (Ratledge, 2004). Therefore, it might be possible, that detoxification of by-products might influence the fatty acid composition of SCO toward shorter fatty acid chain lengths.

Furthermore, yield coefficients for biomass (Y_x/s_) and lipid production (Y_p/s_) were calculated in the presented study to 0.21 g dry biomass per g carbon source and 0.10 g lipid per g carbon source. In comparison, Dillschneider et al. (2014) achieved a higher Y_X/S_ of 0.30 and a slightly higher Y_P/S_ of 0.13 using glucose as sole carbon source, i.e., pure glucose can be converted more efficiently to biomass and lipids as the carbohydrates from fiber hydrolysate which also supports our hypothesis that some energy is needed for detoxification of the fiber hydrolysate Although the lipid content is comparable of pure glucose and fiber hydrolysate cultivation, the much higher biomass concentration in the pure glucose cultivation results in much higher total lipid amount (34.5 g produced lipid with pure glucose vs. 21.5 g produced lipid with fiber hydrolysate). All relevant data of the presented cultivation with cellulose fiber hydrolysate are summarized in Table 1 and compared to the study of Dillschneider et al. (2014). In summary, it was shown that cellulose fiber hydrolysate from organosolv process can be used as a suitable glucose alternative for the lipid production with *C. curvatus*. Cultivating with pure glucose or a mixture of the three sugars present in our hydrolysate in pure form, results in comparable lipid content making the hydrolysate a very promising substrate for an emerging bioeconomy.

**Table 1:** Summary of measured and calculated process parameters of SCO production with *C. curvatus* ATCC 20509 using cellulose fiber hydrolysate from beech wood in comparison to batch cultivations with pure glucose.

	**Cellulose fiber hydrolysate**	Glucose (Dillschneider et al., 2014)^*^
Cultivation time [h]	96	120
Consumed C-source [g]	256.5	Not given
Lipid content [% (w/w)]	47.9	45.3
Dry biomass conc. [g/L]	44.9	76.2
Total lipid amount [g]	21.5	34.5
Y_x/s_ (g biomass per g carbon source)	0.21	0.3
Y_p/s_ (g lipid per g carbon source)	0.10	0.13

In the presented study, produced fatty acids were succeedingly used after transesterification for enzymatic fatty acid sugar ester synthesis in DES. Therefore, a DES consisting of fiber hydrolysate carbohydrates and choline chloride was prepared and characterized.

### Characterizing the DES

To perform a successful biotransformation and for a later optimization of the process, parameters of the DES were identified. In a previous work using the same DES, a pH of 7.5 and water content of 5.4% were already determined (Siebenhaller et al., 2017). In addition to these data, a water activity of 0.101 was measured. The water molecules did not occur in a free form; they are part of the hydrogen bond network of the DES system. Therefore, the water should not be available to perform the hydrolysis of formed sugar ester. To a certain degree, the presence of water molecules may help to maintain the enzymes‘ essential hydrate shell or have other effects on the system like lowering the viscosity. This positive effect to enzymatic reactions was already reported by other groups(Durand et al., 2013; Guajardo et al., 2017).

The viscosity of sugar-based DES is very high and depends on the sugar used, the molar ratio, the water content and the temperature. A similar DES to the one used here, CC:Glu in a molar ratio of 1:1, had a viscosity of 34,400 mPa^*^s at 50°C (Maugeri and Domínguez de María, 2012). The DES is this study consisting of one part CC and one part sugar (glucose and xylose) is less viscous with 2.540 mPa^*^s. A reason could be the water content of 5.4%, which will reduce the viscosity of a DES (Shah and Mjalli, 2014). Further, a nearly 10 times higher viscosity of 23.340 mPa^*^s was determined at 25°C, which also indicates the highly temperature depending viscosity of DES (Maugeri and Domínguez de María, 2012; Stefanovic et al., 2017). The relative low viscosity of the DES may help to increase the mass transfer, which can lead to higher enzymatic reaction yields.

### Detection of synthesized products via thin-layer chromatography

**Figure 3:**
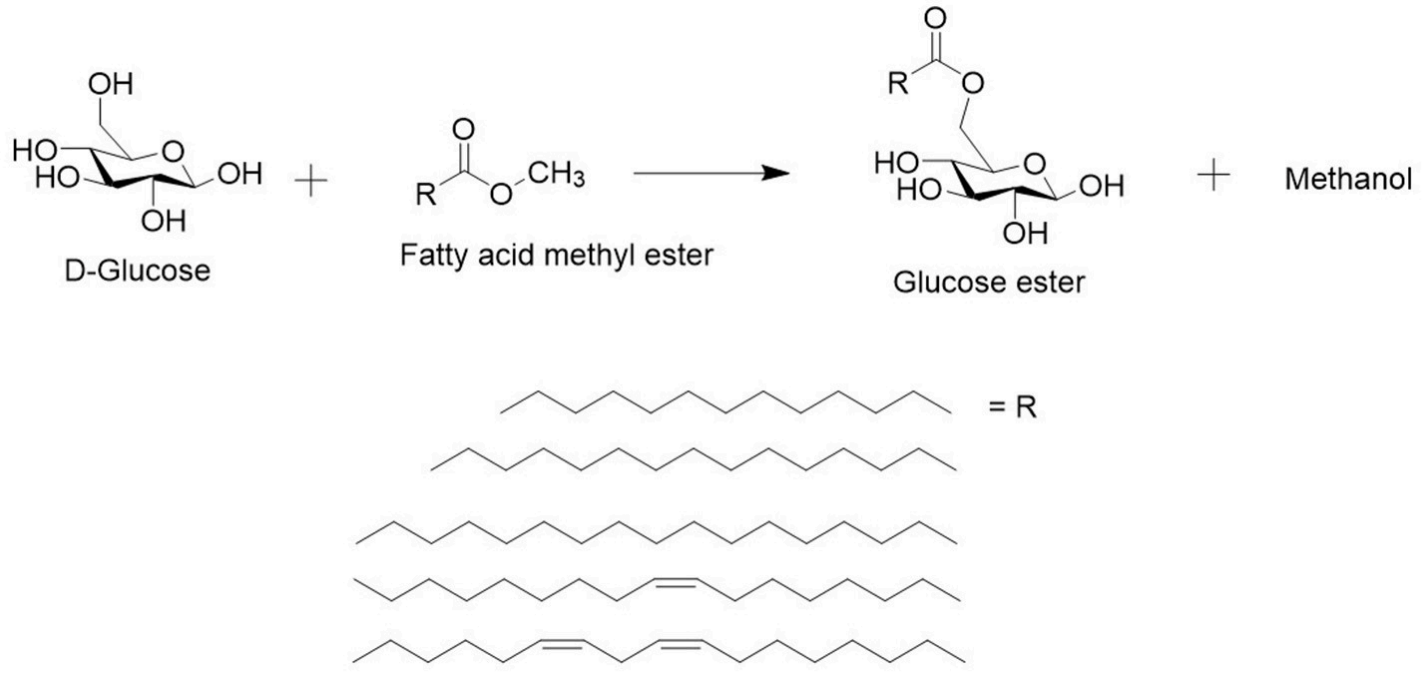
Lipase catalyzed transesterification reaction in the DES with sugar (e.g., glucose) and a methylated fatty acid ester produced from SCO (methyl myristate, methyl palmitate, methyl stearate, methyl oleate and methyl linoleate). The reaction leads to a sugar ester and methanol as side product. Methanol can enter the gas phase and push the reaction forwards or inhibit the enzyme.

**Figure 4:**
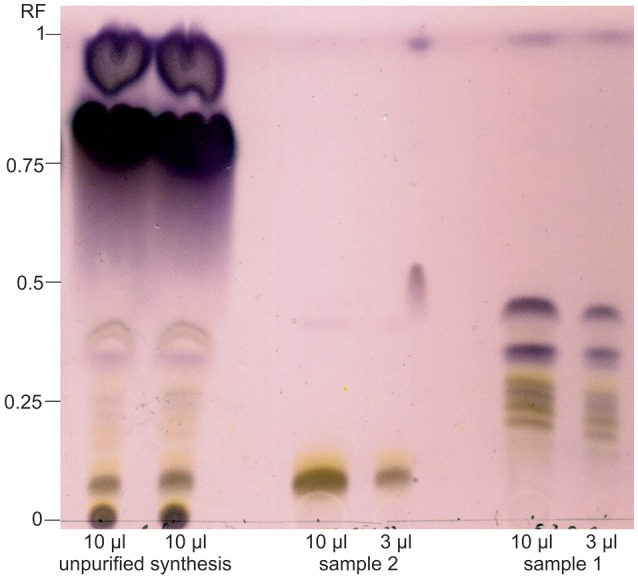
Thin layer chromatography of synthesis products from the enzymatic reaction of microbial fatty acid methyl esters and beech wood cellulose fiber hydrolysate sugars catalyzed by iCalB in a DES reaction system. The crude synthesis extract, the purified and unified sample 1 and 2 were applied; sample 3, as middle fraction of sample 2, was not spotted and direct analyzed by NMR. After the run, the plate was dyed with anise aldehyde solution. Spots with RF 0 represents glucose (dark gray) and xylose (yellow) as leftovers from the DES. The light gray-yellowish spots between RF 0.05 and 0.42 indicate the formation of various fatty acid glucose- and xylose-esters. The high of the spots depends on the sugar, fatty acid chain length and saturation state. The two dark purple spots (RF 0.75 and 0.92) represents fatty acid methyl esters which were not consumed during the synthesis reaction.

For the production of sugar ester, a DES consisting of choline chloride and purified cellulose hydrolysate fraction was prepared and produced FAMEs from SCO and iCalB were added for the synthesis of sugar esters. As a fast analysis of the crude fatty acid sugar ester extract, TLC was performed to detect expected sugar esters (Figure 3). Synthesis products in this extract showed various spots (Figure 4). The spot with the retention factor (RF) 0 is a mixture of glucose (dark gray) and xylose (yellow) of the DES, verified by a standard of the pure sugars (data not shown). The light gray-yellowish spots between RF 0.05 and 0.42 indicate the formation of different glucose- and xylose-esters. The height of each spot depends on the chain length and saturation state of the sugar esters carbonyl chain. In this area, a clear spot with a RF 0.08 seems to be the main product of the enzymatic reaction. Above, two dark purple spots with RF 0.75 and 0.92 are visible. These spots are fatty acid methyl esters which were not consumed during the synthesis reaction.

To get further information and for further analysis, the crude extract was purified and fractionated via flash chromatography. Sample 1 as well as sample 2 show the same spot pattern according to TLC analysis. Therefore, sample 1 and 2 were unified and concentrated. Sample 3 resulted in the clearest spot and was therefore regarded as being the purest fraction of the main product and, consequently, was used for NMR analysis.

In both purified fractions, only trace amounts of FAMEs are visible at the running front. Sample 2 contains the main product, a clear gray colored spot portending to a glucose-ester with a slight yellow aura, indicating for a xylose-ester. The comparison of the height of these spots to known sugar esters led to the assumption that they are sugar-monoesters (Siebenhaller et al., 2016). The analysis of sample 1 showed a gray-yellow striped pattern between RF 0.17 and 0.25. The there-in contained products have either a much longer hydrophobic carbonyl chain, which is unlikely with the FAME-composition applied in our study, or two or more fatty acids are bound to the polar sugar.

During transesterification of a FAME and a sugar, methanol is formed as a side-product. Methanol can enter the gas phase and push the reaction toward acylation but in a sealed reaction vessel and with a boiling point of 64°C, most of the methanol will likely stay in the DES. However, methanol has been reported to inhibit the activity of the lipase (Coulon et al., 1996).

To overcome that, the reaction efficiency might be improved by increasing the temperature to >65°C, taking care that the enzyme remains still active. By using other fatty acids like vinyl esters, acetaldehyde will be formed as side product, which evaporates much easier at lower temperatures (Bornscheuer and Yamane, 1995). However, the vinylation reaction is much more complicated than the methylation of fatty acids. Another method of optimization would be to carry out the process at reduced pressure to remove side products immediately and shift the reaction equilibrium (Ducret et al., 1995).

The successful formation of mono- or polyacylated sugars in this DES system with the used iCalB and commercial available vinyl esters was already shown (Siebenhaller et al., 2017); the synthesis with sustainable fatty acid methyl esters for the production of fatty acid sugar ester was verified by subsequent mass spectrometry and NMR analysis.

### Mass analysis of the formed sugar esters

The successful enzymatic synthesis of sugar esters were affirmed by three different mass analysis procedures. All these methods were necessary to identify clearly the sugar esters, as they have different strengths and weaknesses. First, with the TLC-MALDI-ToF technique it was possible to assign product spots of sample 1 and 2 to a detected *m*/*z* value. In the lane of sample 1, masses were detected in three areas. Surprisingly, in area 1 (A1: RF 0.59–0.67) and area 2 (A2: RF 0.72-.082) the same *m*/*z* values were determined (Supplement 2). Several *m*/*z* values of sodium adducts of glucose-di-esters with the following fatty acid pairs were identified: *m*/*z* 680.04 (myristic acid/stearic acid or more likely palmitic acid/palmitic acid), *m*/*z* 706.47 (palmitic acid/oleic acid), *m*/*z* 708.09 (palmitic acid/stearic acid), *m*/*z* 727.66 (linoleic acid/linoleic acid) and *m*/*z* 734.10 (stearic acid/oleic acid). Throughout the entire area 3 (A3: RF 0.86–0.88), only *m*/*z* 686.0 was detected, which corresponds to a protonated glucose, acylated with palmitic acid and stearic acid. Due to the high number of potentially formed sugar-di-esters, more possible combinations are feasible. So it is possible, that *m*/*z* 680.04 corresponds to a protonated xylose, which is linked to 2 oleic acids, or to stearic acid/linoleic acid.

By applying sample 2 onto the silica plate and subsequent analysis, two mass areas (a1; RF 0.33–0.45 and a2: RF 0.84–0.89) were found. In a1, sodium adducts of the more polar reaction products glucose palmitate (*m*/*z* 441.66) glucose oleate (*m*/*z* 467.71) and glucose stearate (*m*/*z* 469.74) were detected. a2 indicates the presence of more hydrophobic glucose-di-esters, which was expected due to the TLC separation. One of these di-ester corresponds to a protonated glucose with palmitic acid and stearic acid (m/z 686.04), the other di-ester matched to the sodium adduct of a glucose, linked to 2 linoleic acids (*m*/*z* 727.61). These results show that the enzymatic synthesis was successful.

Due to possible matrix and silica problems by the detection of molecules with low masses, ESI-Q-ToF MS were performed to verify the results.

By applying ESI-Q-ToF MS to sample 2 various sugar-mono-esters were identified. The synthesis of glucose palmitate (M_GP_ = 418.29 Da) was verified through the occurrence of ions at *m*/*z* 441.27 (M_GP_ + Na^+^), *m*/*z* 436.30 (M_GP_ + ${\text{NH}}_{4}^{+}$), *m*/*z* 401.26 (M_GP_-H_2_O + H^+^) and *m*/*z* 383.23 (M_GP_-2H_2_O + H^+^). The same adducts of glucose oleate (M_GO_ = 444.31 Da) were detected at *m*/*z* 467.27, 462.32, 427.26 and 409.27. Further, glucose stearate (M_GS_ = 446.32 Da) and glucose linoleate (M_GL_ = 442.29 Da) was detected through the ions M_GS_-H_2_O + H^+^ (*m*/*z* 429.29) respective M_GL_-H_2_O + H^+^ (*m*/*z* 425.25). Thus, it was possible to detect sugar esters of the most common FAMEs.

In addition, a MALDI-ToF MS of sample 1 was accomplished to verify the occurrence of sugar-di- or poly-ester with higher masses, which couldn't be detected via ESI-Q-ToF MS and to overcome possible problems of the TLC-silica. There, masses corresponding to synthesized sugar-di-esters were detected, too. Sodium adducts with *m*/*z* 733.56, *m*/*z* 731.55, *m*/*z* 705.52, and *m*/*z* 679.55 indicates the formation of glucose-di-ester with the following acylated fatty acid pairs: stearic acid/oleic acid (712.58 Da), stearic acid/linoleic acid (708.55 Da), oleic acid/oleic acid (708.55 Da), palmitic acid/oleic acid (682.53 Da) and palmitic acid/palmitic acid (656.52 Da). At *m*/*z* 699.5, the sodium adducts of a di-acylated xylose with oleic acid/linoleic acid (676.53 Da) shows, that xylose was used as a substrate, too.

The results of the mass analysis confirmed the formation of sugar esters in the used DES with FAMEs.

Beside the mentioned products, other sugar-mono- and di-esters might be formed. Since it is shown in literature, it cannot be excluded that sugar-poly-ester were also synthesized in this reaction system, even if none were detected (Siebenhaller et al., 2016). Since many products or their adducts have the same mass, it is nearly impossible to distinguish between them. It can only be stated that, based on the FAME-mixture used and the amount of fatty acids contained therein, some sugar esters will be probably less common than others. Sugar esters with the four main fatty acids (palmitic acid, stearic acid, oleic acid and linoleic acid) were detected; therefore, the used FAME-Mix is a suitable substrate for this reaction.

It was possible to identify some xylose esters in fraction 1 via MALDI-ToF MS, but these are much rarer than glucose ester. Since glucose occurs 4-times more often than xylose in the purified and dried cellulose fraction, this is justified.

Mass spectra are shown in the Supplement 3, 4.

### Identification of sugar ester structure by NMR

The purified and fractionated sample 3 of an iCalB catalyzed synthesis reaction in a DES, based on lignocellulosic sugars, with FAMEs, was investigated with one and two-dimensional ^1^H and ^13^C NMR spectra. Glucose was identified through one clear major carbohydrate system, starting from the anomeric protons of the ^1^H COZY and ^13^C HMBC spectra. Cross peaks between the two protons attached at the glucoses‘ C6 at 4.23 and 4.35 ppm with the lipid carbonyl at 174.03 ppm in the ^1^H^13^C HMBC indicates, that glucose was acylated (Table 2). Due to some impurities and the used mixture of fatty acid methyl esters, it was not possible to clearly identify which FAME is connected to the glucose molecule.

**Table 2:** Chemical shifts of the main product, present in the purified and fractionated sample 3.

**Glucose**	**C shift (ppm)**	**H shift (ppm)**
-C^1^H-O-	92.6	5.21
-C^2^H-	72.6	3.47
-C^3^H-	74.0	3.76
-C^4^H-	70.2	3.36
-C^5^H-	69.6	3.97
-C^6^H- (acylated C′174.03)	63.7	4.23
-C^6^′H- (acylated C′174.03)	63.7	4.35

*The shifts indicate a sugar system, identified as glucose with acylation at the C6-atoms. 8 line number 464)*.

A view on the structure of glucose shows, that less steric effects affect the glucoses exposed C6-atom, so that this acylation site seems to be favored. This was also observed in previous work by our group (Siebenhaller et al., 2017).

This result and the mass analysis data confirm that sugar esters have been produced during the synthesis process.

However, in the analyzed sample no traces of xylose or acylated xylose were detected. This can be explained by the fact that xylose occurs over 4 times less frequently than glucose and that xylose esters may have been removed during purification of the analyzed fraction. However, in the mass analysis of unified fraction 2, xylose or its esters were not detected either.

## Conclusion

The presented study demonstrates the possibility to use sustainable lignocellulosic biomass as sole substrate for the production of high valuable fatty acid sugar esters. It was shown, that cellulose fiber hydrolysate from organosolv process is a suitable substrate for SCO production with *C. curvatus* being completely comparable to pure sugars regarding biomass and lipid content. Subsequently, a DES reaction system based on sugars of the hydrolysate was established, acting simultaneously as reaction media and together with the produced fatty acids as substrate for the enzymatic sugar ester synthesis. The successful formation of sugar esters were proven by NMR analysis. Further, several sugar (di-)esters like glucose palmitate or oleate and glucose di- linoleic acid were detected by various MS experiments. For a better understanding of the reaction media characteristics of the used DES were determined.

## Author contributions

SS performed experiments, conceived the work, worked on coordination of experiments and supervised JK. SS also wrote the enzymatic part of the manuscript and contributed to proofreading. JK substantial performance in all experiments and helped revise the final version. FK and GB-W performed the ESI-Q-ToF MS and MALDI-ToF MS measurements, wrote parts of the MS-section and critically revised the manuscript. CM-G and BL execution and evaluation of the NMR analysis and revision of the manuscript. FH, TH, and SZ prepared and analyzed the beech wood cellulose fiber hydrolysate. They also performed the TLC-MALD-ToF MS experiments and provided critical proofreading of the manuscript. CS critical revision of the work and manuscript and important intellectual content. KO coordination of experiments and supervised JK. Also contributed to the intellectual content, the writing of the main part of the SCO fermentation part of the manuscript and proofreading.

### Conflict of interest statement

The authors declare that the research was conducted in the absence of any commercial or financial relationships that could be construed as a potential conflict of interest.
