# Supplementary material for: Integrated Process for the Enzymatic Production of Fatty Acid Sugar Esters Completely Based on Lignocellulosic Substrates
Source: Front Chem. 2018 Sep 13;6:421. doi: 10.3389/fchem.2018.00421 (PMC6146371; doi:10.3389/fchem.2018.00421)
Supplement: Supplementary file 2 [file Data_Sheet_2.PDF]

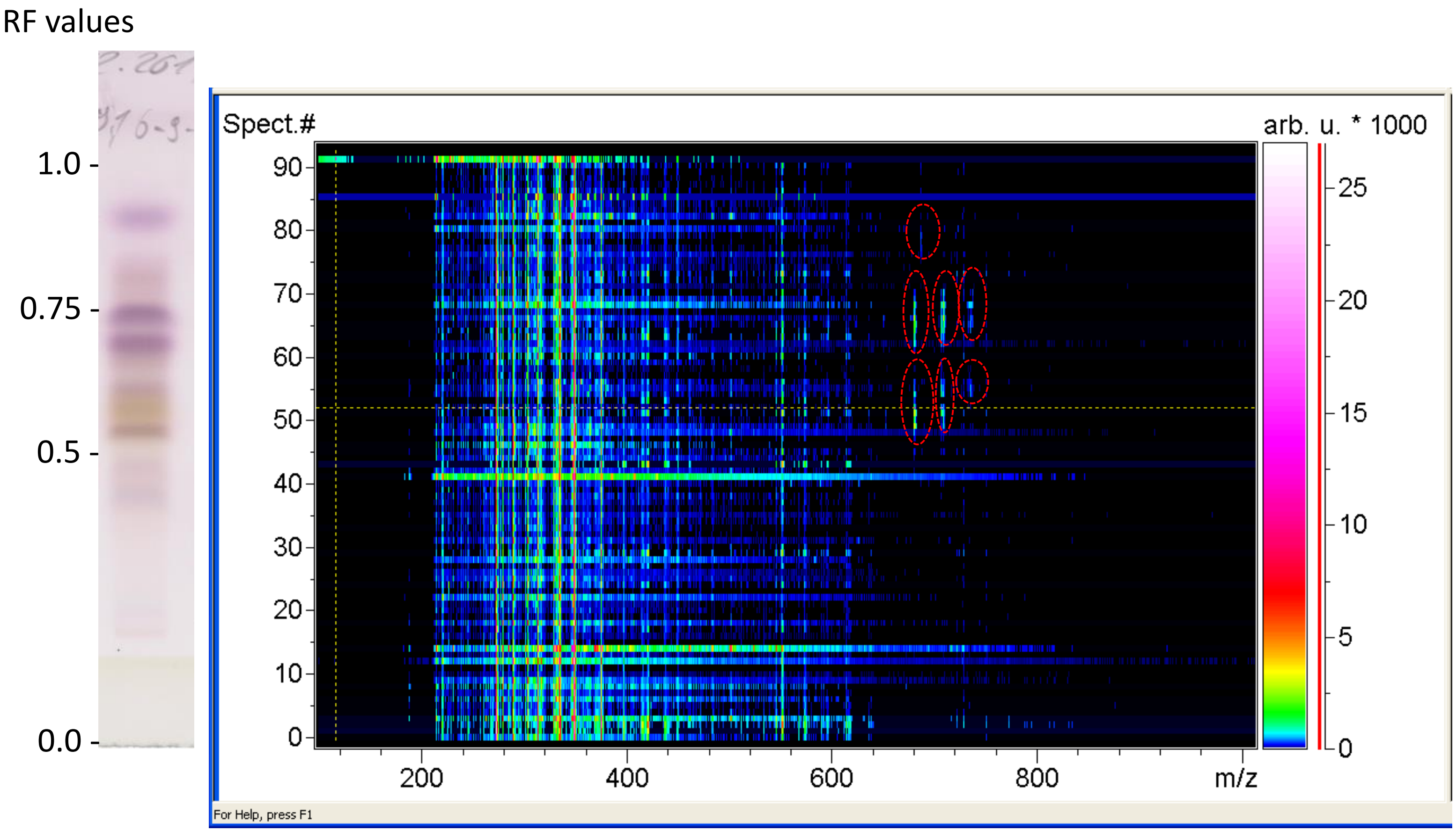

Supplement 2: TLC-MALDI-ToF MS of fraction 6-9

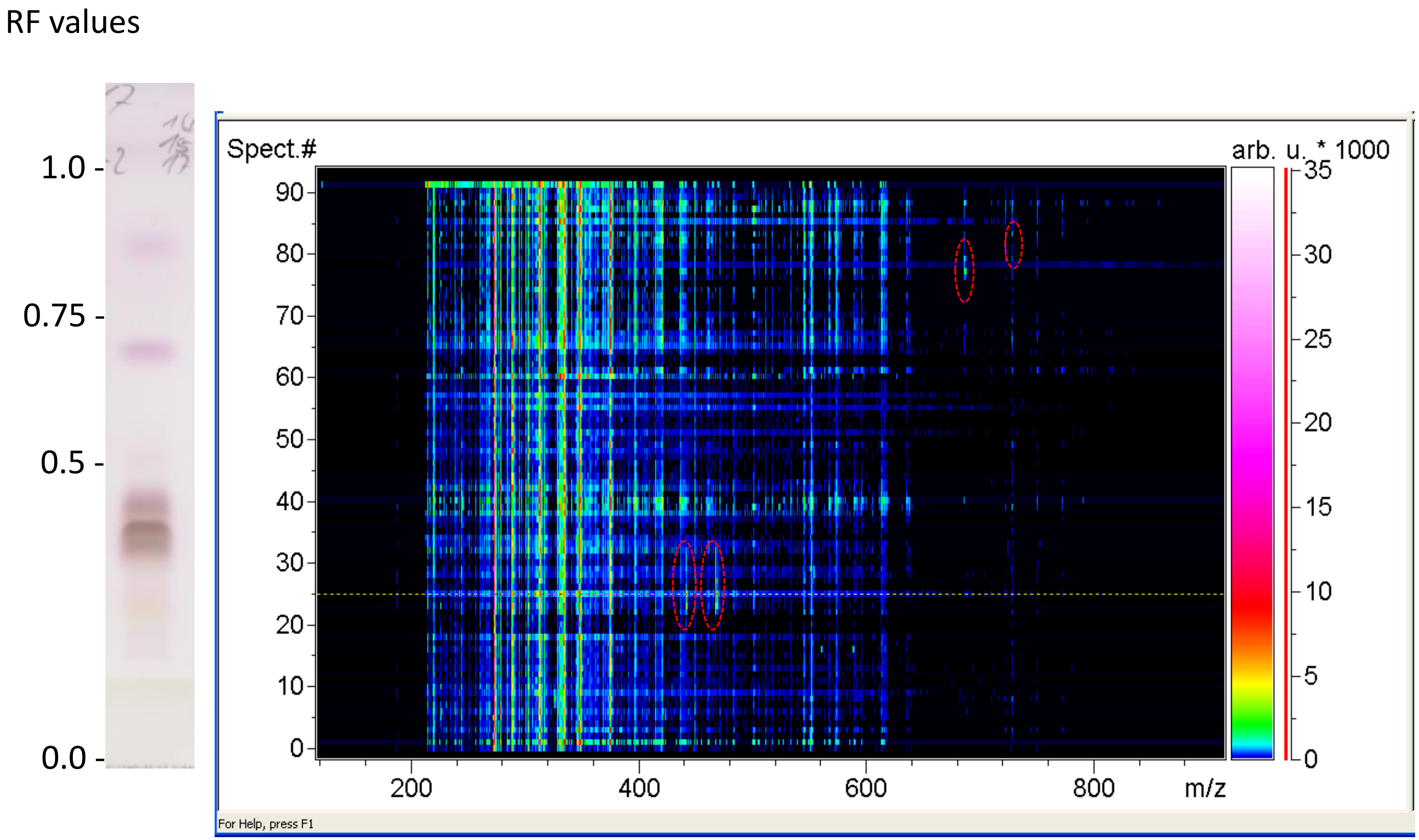

Supplement 1: TLC-MALDI-ToF MS of fraction 14,15,17
