# Supplementary material for: Integrated Process for the Enzymatic Production of Fatty Acid Sugar Esters Completely Based on Lignocellulosic Substrates
Source: Front Chem. 2018 Sep 13;6:421. doi: 10.3389/fchem.2018.00421 (PMC6146371; doi:10.3389/fchem.2018.00421)
Supplement: Supplementary file 3 [file Data_Sheet_3.PDF]

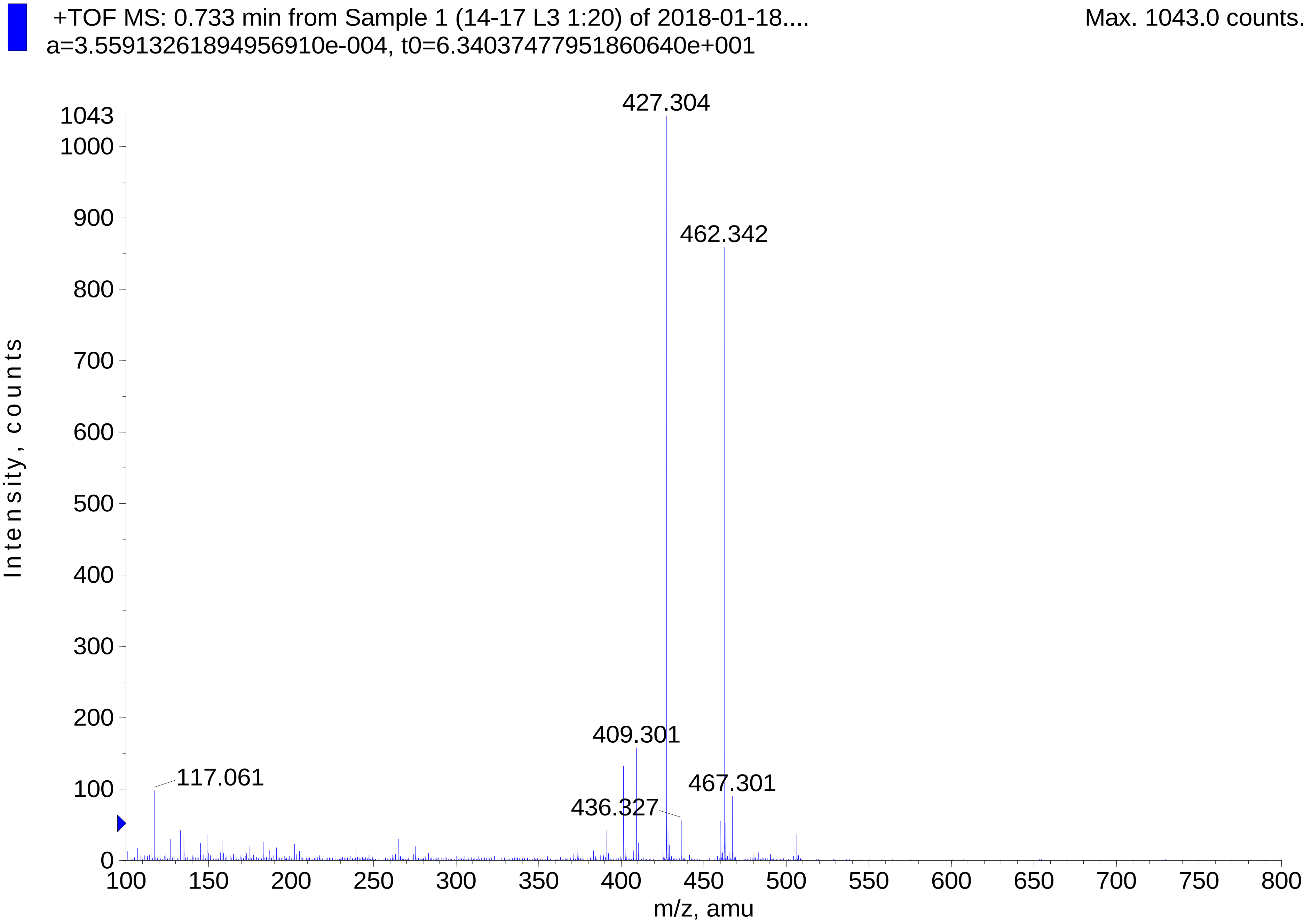

Supplement 3: ESI-Q-ToF MS of fraction 14+15+17

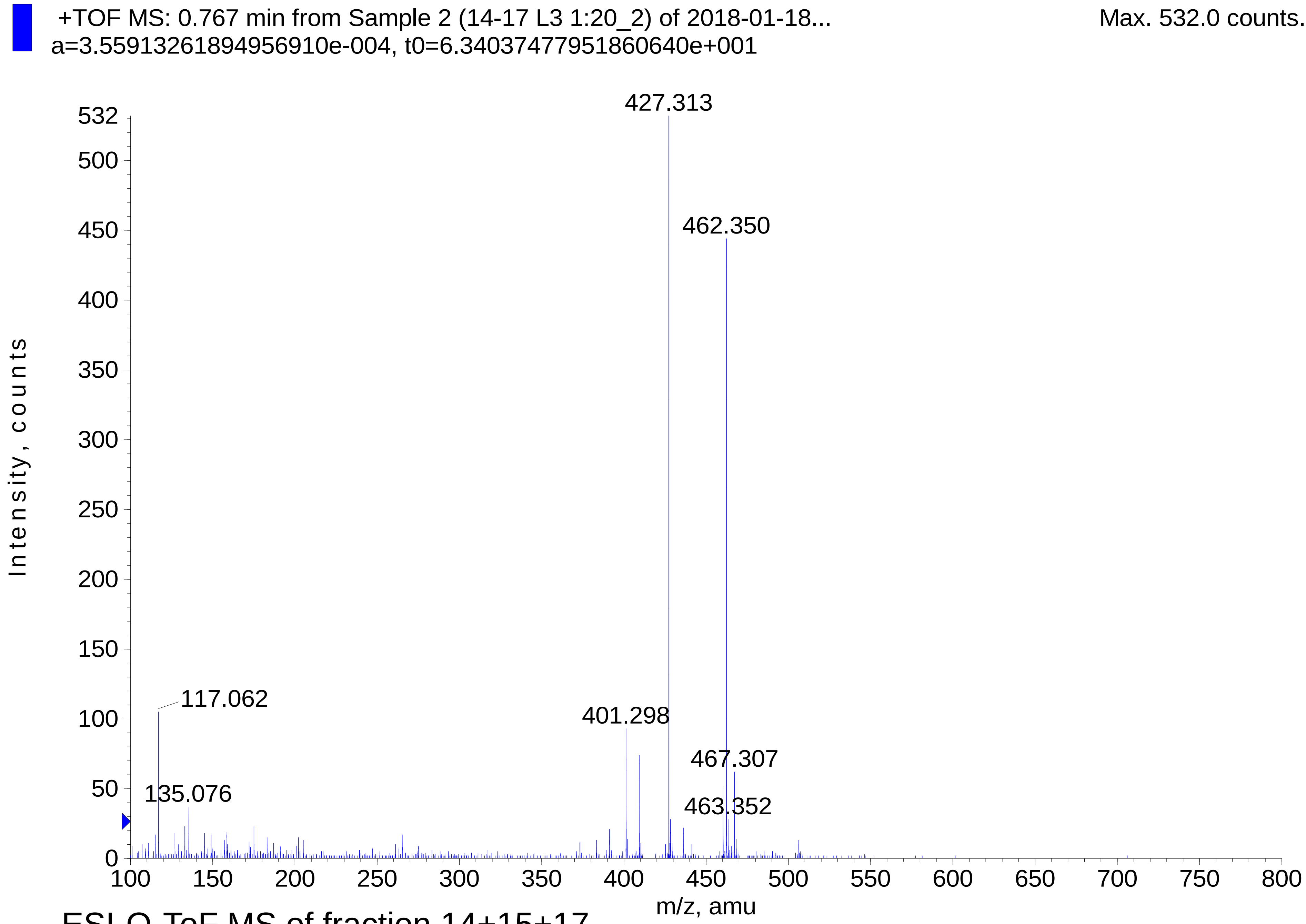

ESI-Q-ToF MS of fraction 14+15+17

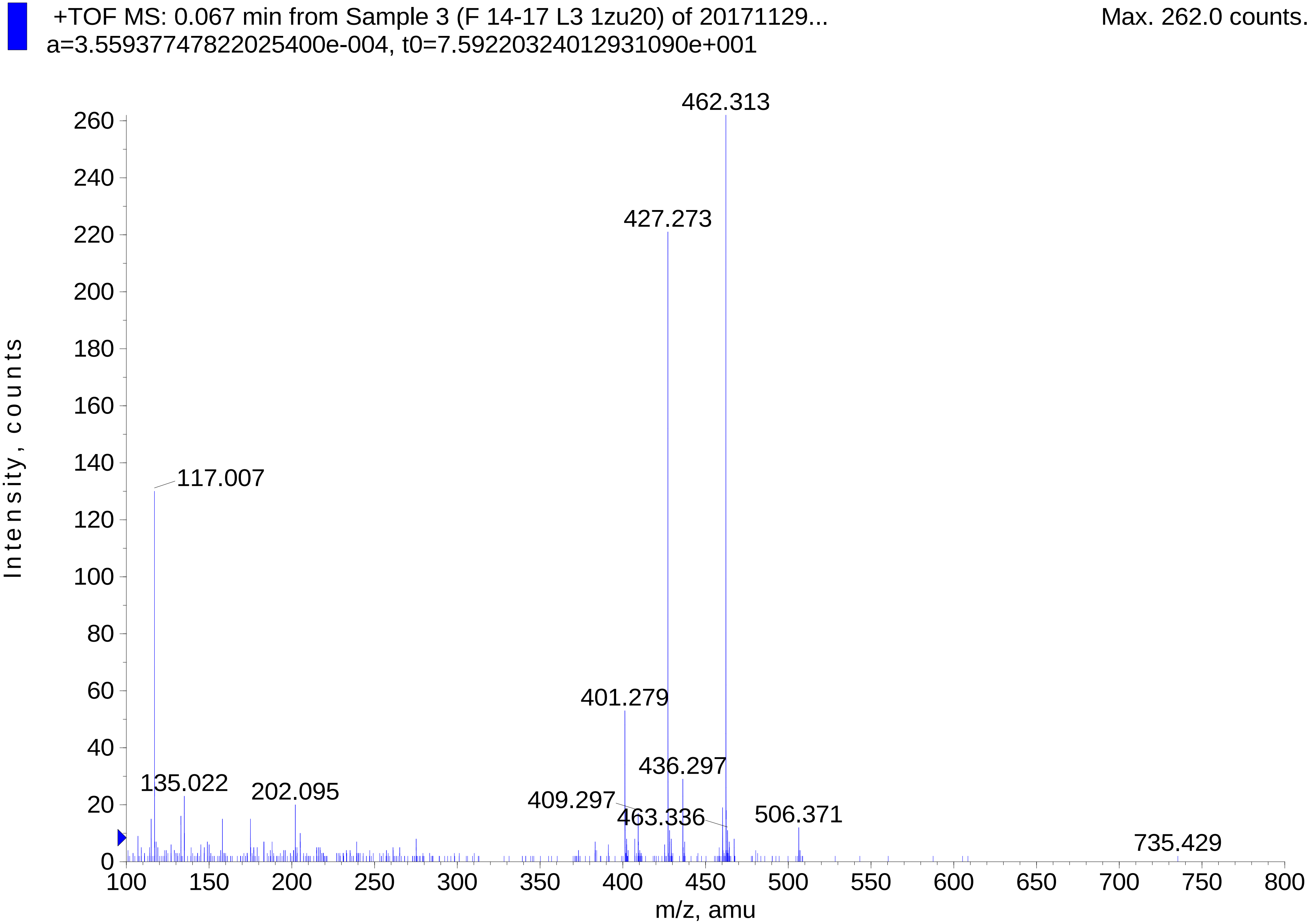

ESI-Q-ToF MS of fraction 14+15+17

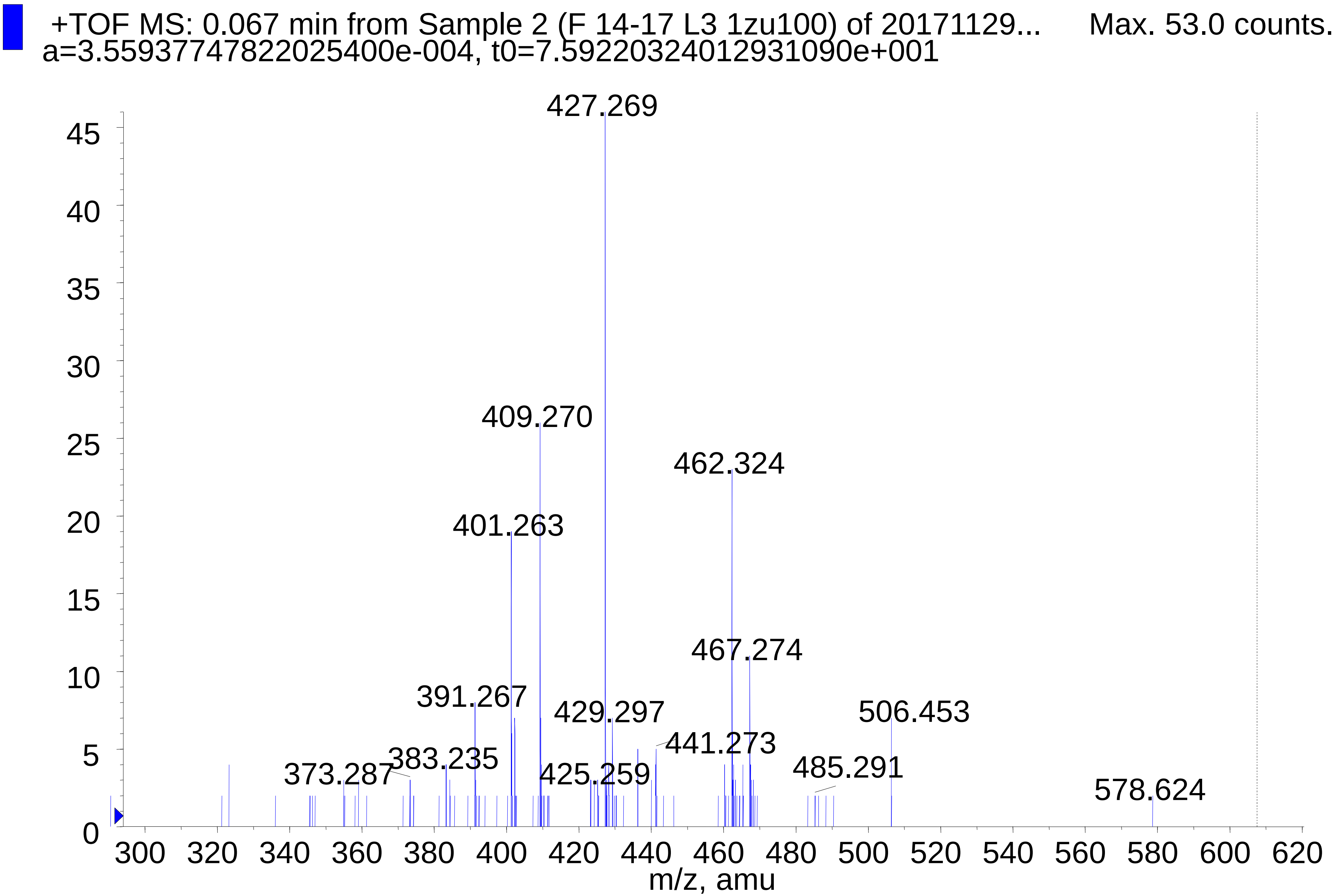

ESI-Q-ToF MS of fraction 14+15+17
