# Supplementary material for: Integrated Process for the Enzymatic Production of Fatty Acid Sugar Esters Completely Based on Lignocellulosic Substrates
Source: Front Chem. 2018 Sep 13;6:421. doi: 10.3389/fchem.2018.00421 (PMC6146371; doi:10.3389/fchem.2018.00421)
Supplement: Supplementary file 4 [file Data_Sheet_4.PDF]

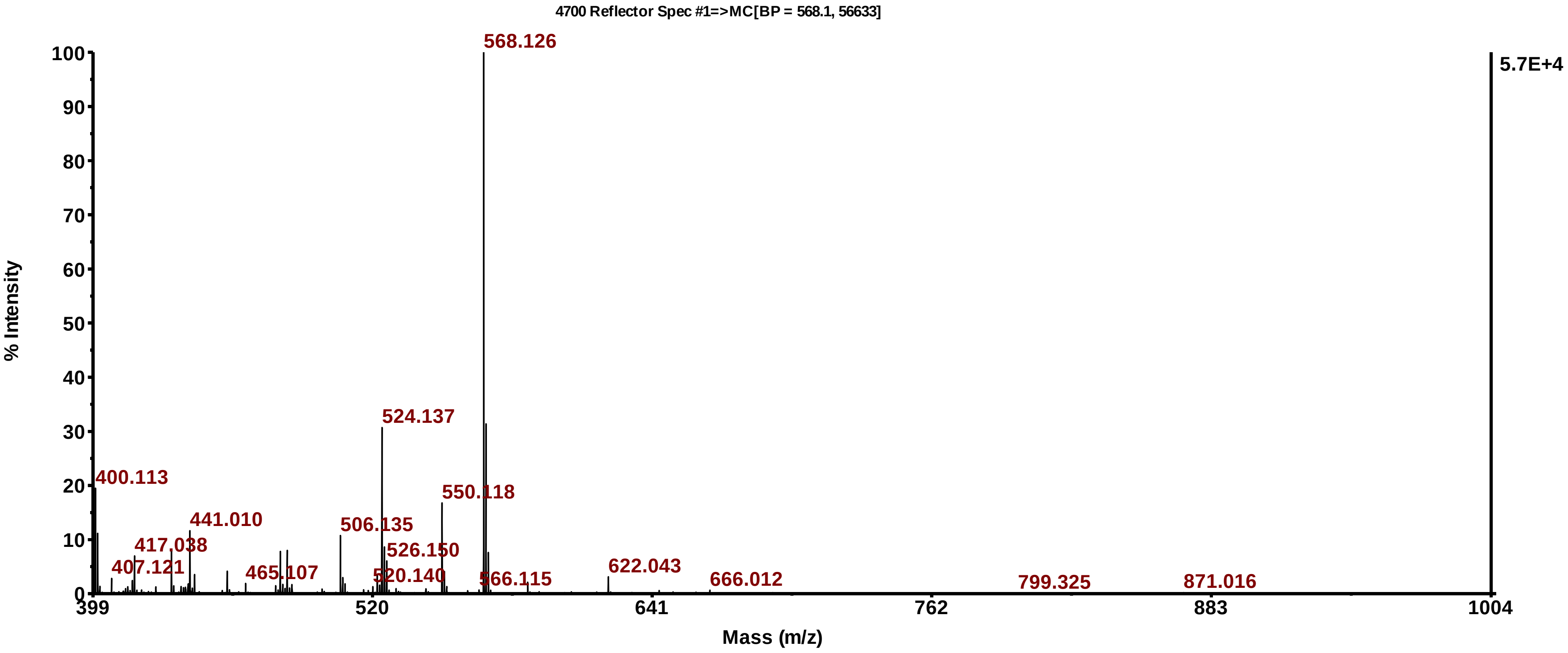

Supplement 4: CHCA Blank via MALDI-ToF MS

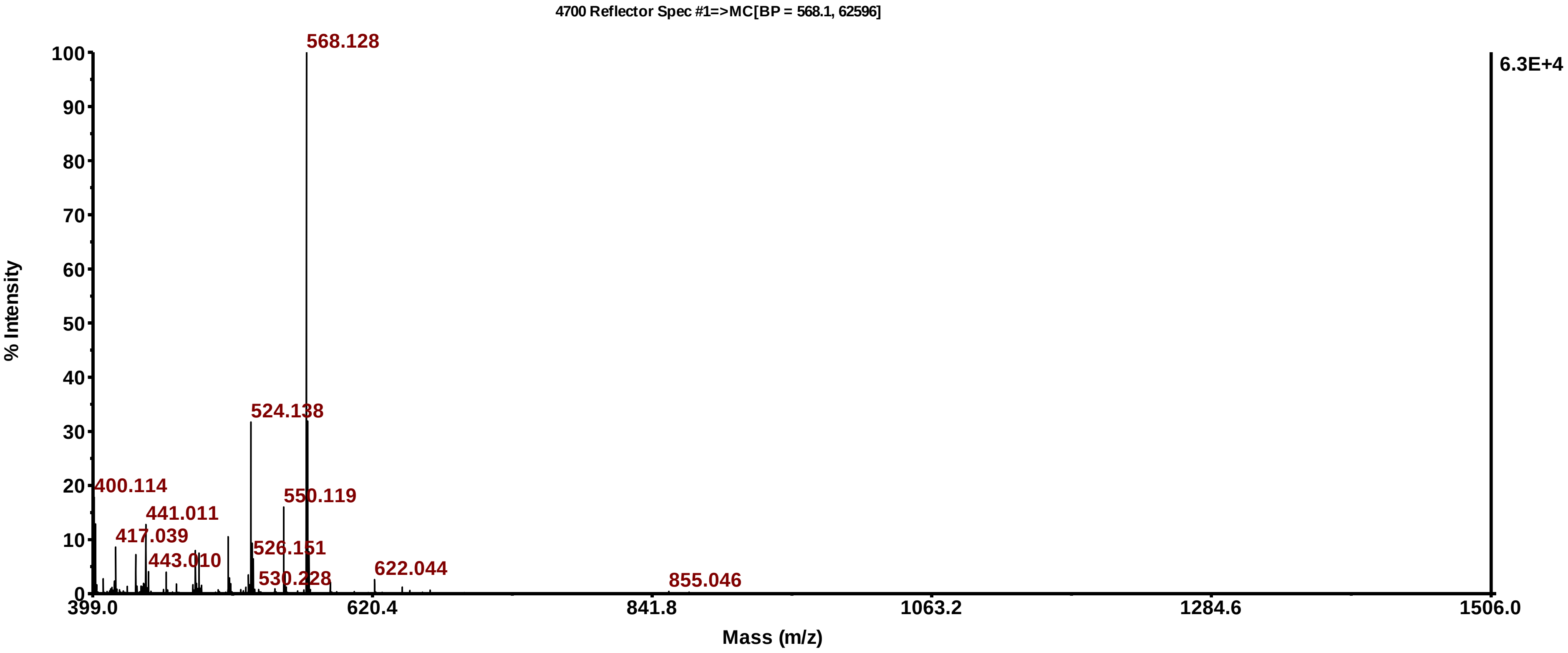
4700 Reflector Spec #1=>MC[BP = 568.1, 62596]
568.128
524.138
400.114
550.119
441.011
417.039
526.151
443.010
530.228
622.044
855.046
6.3E+4
% Intensity
Mass (m/z)
399.0
620.4
841.8
1063.2
1284.6
1506.0

CHCA Blank

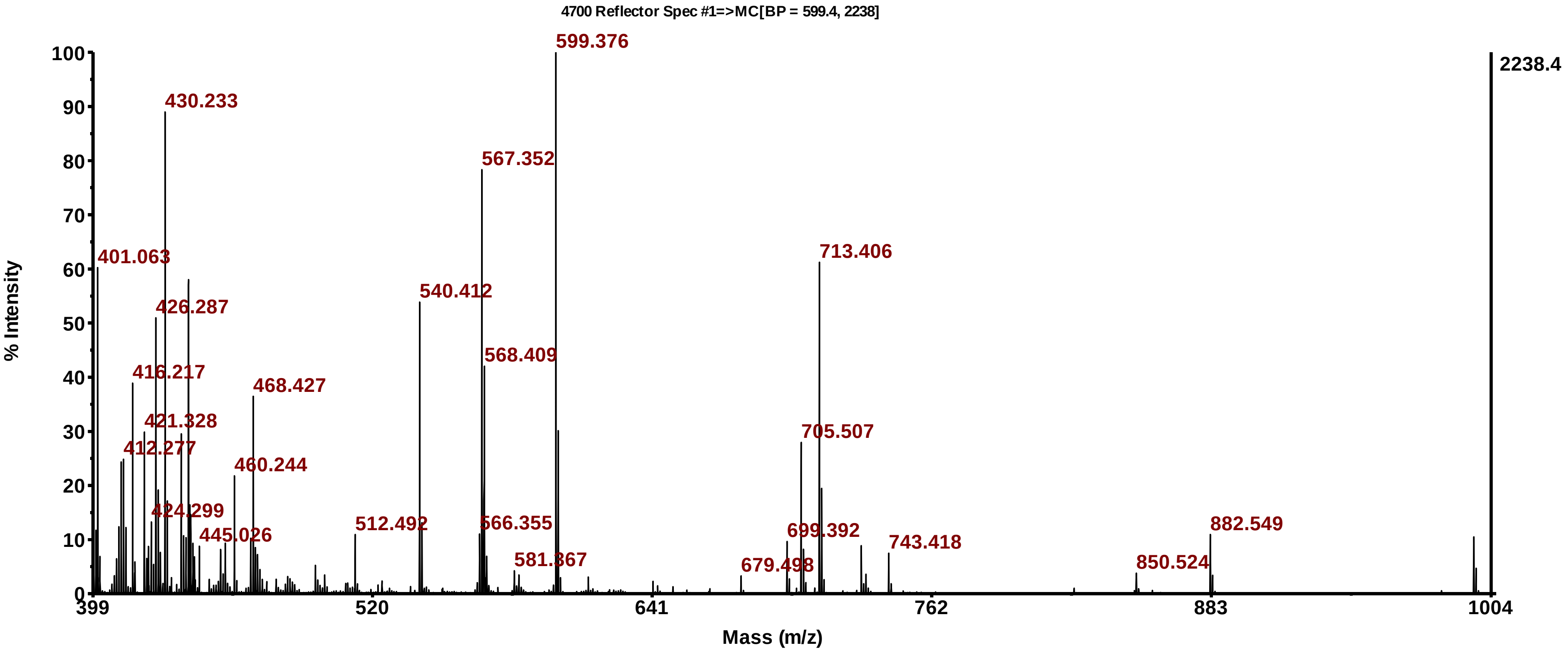
4700 Reflector Spec #1=>MC[BP = 599.4, 2238]
% Intensity
Mass (m/z)
2238.4
599.376
430.233
567.352
401.063
713.406
540.412
426.287
568.409
416.217
468.427
421.328
705.507
412.277
460.244
424.299
445.026
512.492
566.355
699.392
743.418
882.549
581.367
679.498
850.524
399
520
641
762
883
1004

Sample 6-9

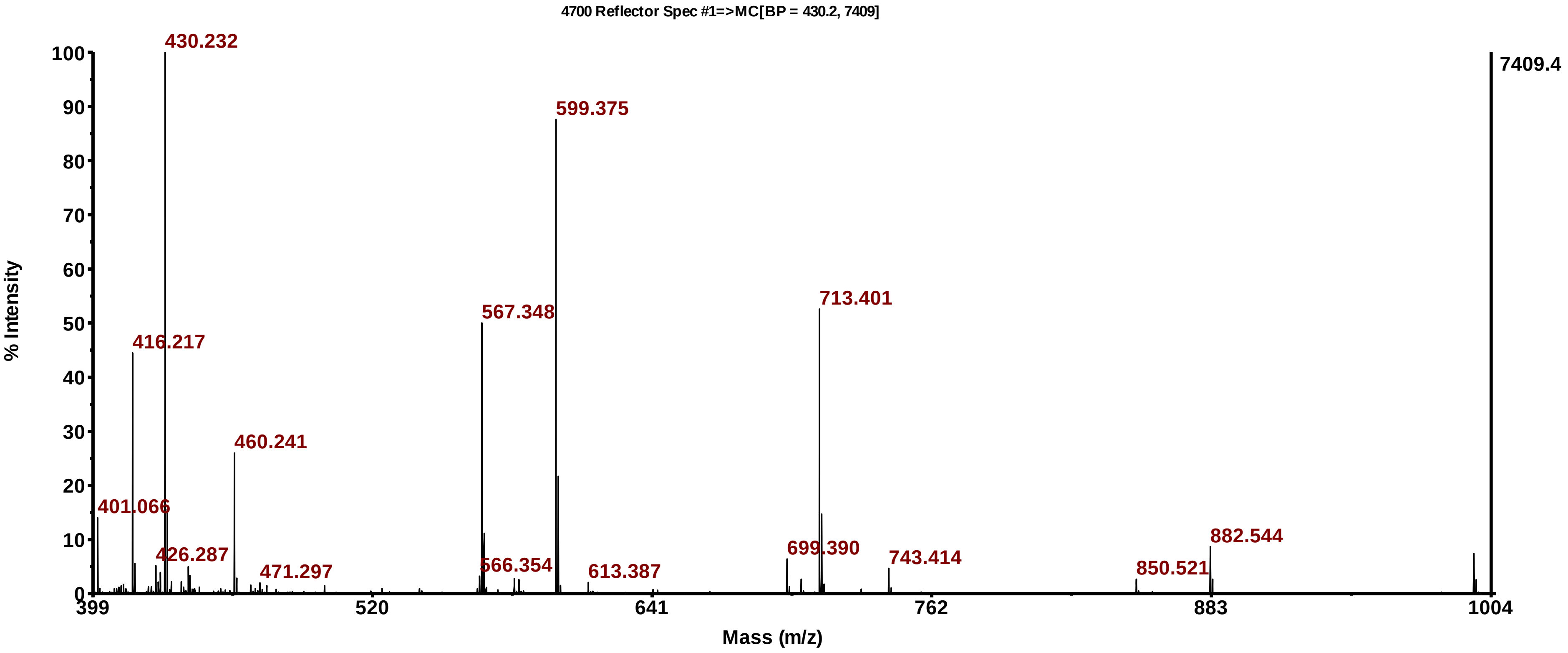

4700 Reflector Spec #1=>MC[BP = 430.2, 7409]
% Intensity
100
90
80
70
60
50
40
30
20
10
0
430.232
7409.4
599.375
713.401
567.348
416.217
460.241
401.066
882.544
699.390
426.287
743.414
566.354
850.521
471.297
613.387
399
520
641
762
883
1004
Mass (m/z)

Sample 6-9

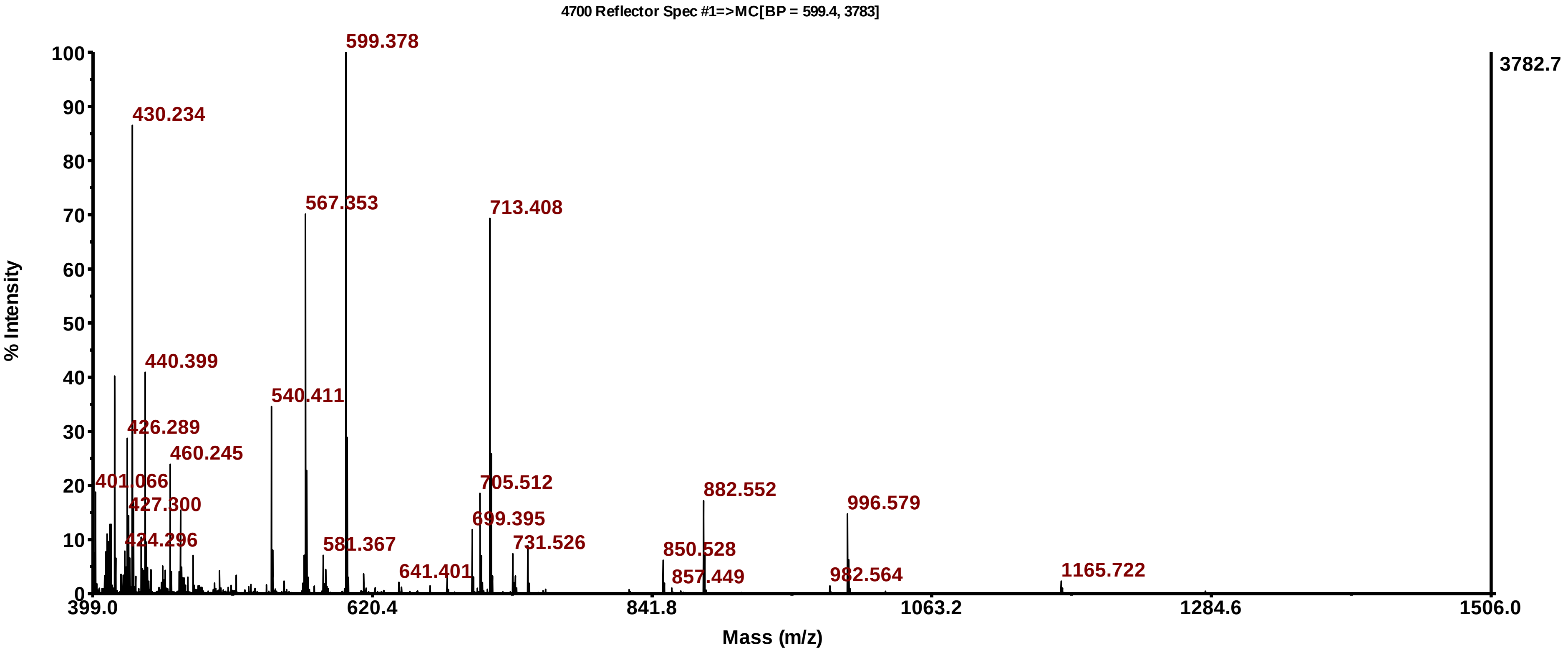
4700 Reflector Spec #1=>MC[BP = 599.4, 3783]
% Intensity
3782.7
599.378
430.234
567.353
713.408
440.399
540.411
426.289
460.245
401.066
705.512
882.552
427.300
996.579
699.395
424.296
581.367
731.526
850.528
641.401
857.449
982.564
1165.722
399.0
620.4
841.8
1063.2
1284.6
1506.0
Mass (m/z)

Sample 6-9

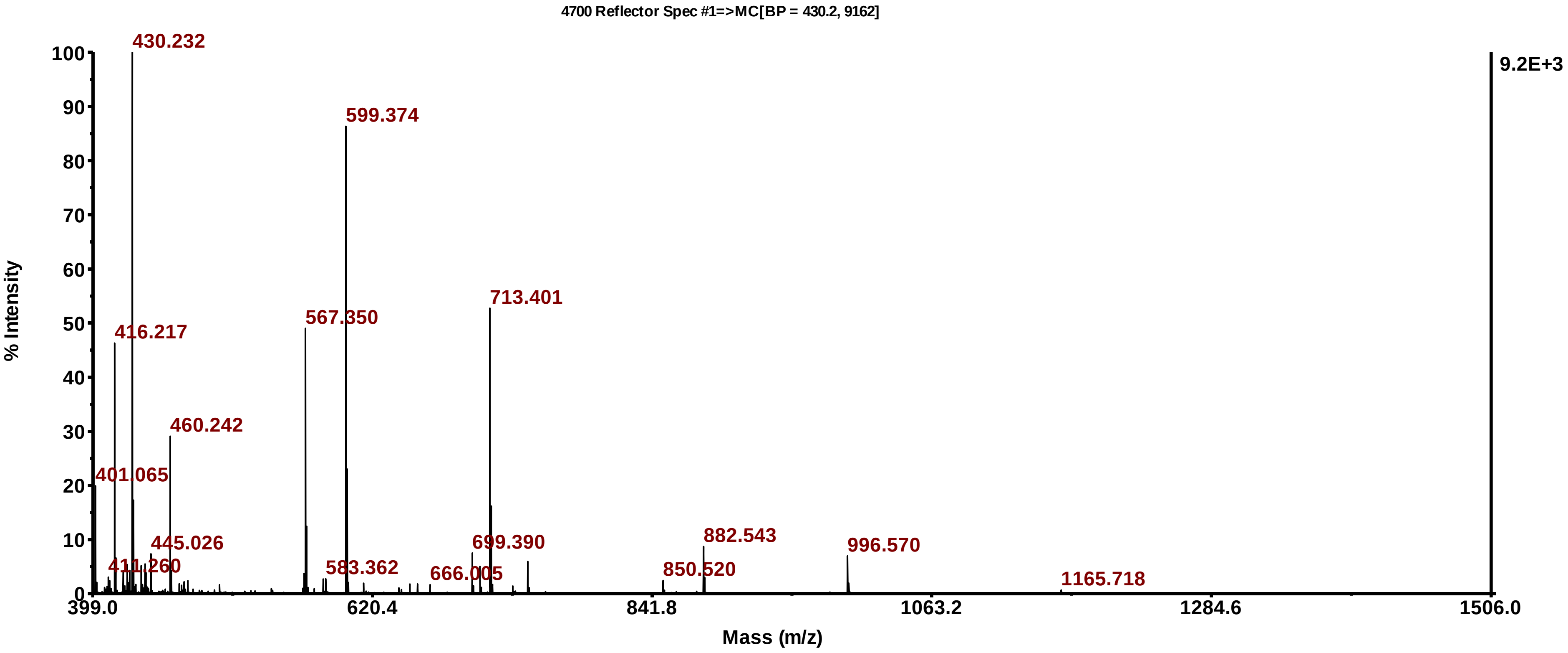
4700 Reflector Spec #1=>MC[BP = 430.2, 9162]
% Intensity
Mass (m/z)
9.2E+3
430.232
599.374
713.401
567.350
416.217
460.242
401.065
445.026
411.260
583.362
666.005
699.390
882.543
850.520
996.570
1165.718
399.0
620.4
841.8
1063.2
1284.6
1506.0
100
90
80
70
60
50
40
30
20
10
0

Sample 6-9
